# Kidney sparing surgery versus radical nephroureterectomy in upper tract urothelial carcinoma: a meta-analysis and systematic review

**DOI:** 10.3389/fonc.2025.1448079

**Published:** 2025-04-02

**Authors:** Leqing Zhou, Chuyang Huang, Sheng Sun, Keping Ning, Shan Tang

**Affiliations:** Department of Urology, Shaoyang Hospital affiliated to University of South China, Shaoyang, China

**Keywords:** upper tract urothelial carcinoma, kidney-sparing surgery, segmental urethrectomy, endoscopic management, survival outcomes, renal function, meta-analysis

## Abstract

**Objective:**

Kidney-sparing surgery (KSS) has been increasingly performed in patients with upper tract urothelial carcinoma (UTUC) in recent years. We aim to conduct a systematic review and meta-analysis comparing the long-term oncologic and renal function outcomes of KSS with those of radical nephroureterectomy (RNU) for UTUC.

**Materials and methods:**

A literature search was conducted on PubMed, Embase, and Web of Science in January 2024. A meta-analysis was performed to analyze overall survival (OS), cancer-specific survival (CSS), recurrence-free survival (RFS), intravesical recurrence-free survival (IVRFS), metastasis-free survival (MFS), and surgery-related estimated glomerular filtration rate (eGFR) variations.

**Results:**

A total of 32 studies with 21615 patients were included in this meta-analysis. Patients treated with KSS were less likely to have hydronephrosis, more often had low-grade tumors, and were more frequently at a low-stage compared to those undergoing RNU. There were no significant differences between the KSS and RNU groups in terms of 5-year OS, 5-year CSS, 5-year RFS, 5-year IVRFS, 5-year MFS, and hazard ratios (HRs) for OS and CSS based on univariate or multivariable Cox regression analysis. Similar results were found in subgroup analyses comparing segmental urethrectomy (SU) with RNU. In the comparison between the endoscopic management (EM) and RNU groups, EM was associated with worse overall survival outcomes (HR,1.40; 95%CI,1.08-1.82; P=0.01) based on multivariable Cox regression analysis, and the upper tract recurrence rate (OR,39.06; 95%CI, 14.55-104.85; P<0.00001) was significantly higher in the EM group. On the other hand, in patients treated with KSS, postoperative renal function as measured by eGFR increased by 0.4ml/min/1.73 m^2^, while it decreased by 11.4ml/min/1.73 m^2^ in the RNU group (WMD, 11.81 ml/min/1.73 m^2^; 95%CI,9.06-14.56; P<0.0001).

**Conclusion:**

Our meta-analysis supports similar oncological outcomes between KSS and RNU, although tumor characteristics were not equally balanced. KSS can be considered the best choice option for patients with low-risk UTUC, as it offers better preservation of renal function. In terms of kidney-sparing options, SU could be a better alternative for the treatment of ureteral tumors compared to ureteroscopy, due to the higher local recurrence rate associated with EM.

**Systematic review registration:**

https://inplasy.com/inplasy-2024-5-0051/, identifier (INPLASY202450051).

## Introduction

Upper tract urothelial carcinoma (UTUC) is a relatively rare cancer that accounts for 5-10% of all urothelial carcinomas affecting the renal pelvis and ureter (1). Regardless of tumor location, radical nephroureterectomy (RNU) with bladder cuff excision is still considered the standard treatment for UTUC. However, RNU has a significant and enduring detrimental impact on renal function, which puts the patient at risk of chronic kidney disease (CKD) and related sequelae, particularly in patients with impaired renal function (2). Kidney-sparing surgery (KSS) has been proposed as an alternative to RNU for the treatment of selected cases of UTUC, including ureteroscopy, percutaneous access, and segmental urethrectomy (SU). The European Association of Urology (EAU) guidelines recommend KSS as a treatment option for UTUC patients with low-grade, low-stage, unifocal, or small-volume tumors (≤ 2 cm) (3). Patients with compelling indications such as solitary kidney, renal insufficiency, or bilateral UTUC can also be considered KSS as a treatment for UTUC.

However, the safety and efficacy of KSS are still controversial in some literature, the aim of this study is to perform a systematic review and meta-analysis on the oncologic and renal function outcomes between KSS and RNU for patients with UTUC.

## Materials and methods

### Search strategy

Two investigators (LQZ and SS) performed a computerized bibliographic search using a combined text and MeSH heading search strategy in Pubmed, Embase, and Web of Science up to January 2024. The following search strategy was used for this study: (Ureteroscopic OR Percutaneous Nephroscopy OR Endoscopy OR Segmental Ureterectomy OR Partial Ureterectomy OR Distal Ureterectomy OR Nephron Sparing OR Kidney Sparing) AND (Upper Tract Urothelial Carcinoma OR Urothelial Carcinoma OR Transitional Cell Carcinoma) AND (Radical OR Nephroureterectomy). In addition, the reference lists in recent reviews, meta-analyses, and included articles were manually searched to identify related articles. The research was focused on English-language studies and did not include conference abstracts, conference papers, notes, letters, editorials, and short surveys.

### Inclusion and exclusion criteria

According to the Preferred Reporting Items for Systematic Reviews and Meta-analysis (PRISMA) guidelines, we used the Population, Intervention, Comparator, Outcome (PICO) approach to define study eligibility: patients with UTUC (P) undergoing KSS (I) or RNU (C) to compare oncological and renal function outcomes (O). We defined the inclusion and exclusion criteria for study selection at the initiation of the search. The inclusion criteria were: (1) Patients diagnosed with non-metastatic upper tract urothelial carcinoma; (2) The intervention group included only patients treated with ureteroscopic or percutaneous surgery, SU, or distal urethrectomy; (3) The control group included only patients treated with RNU; (4) The study reported at least one of the following outcomes: overall survival (OS), cancer-specific survival (CSS), recurrence-free survival (RFS), intravesical RFS (IVRFS), metastasis-free survival (MFS), and changes in estimated glomerular filtration rate (eGFR) related to surgery. The exclusion criteria were: (1) Single-arm studies; (2) Metastatic UTUC; (3) Adolescents (under 18 years of age); (4) The study period in the literature before 1990.

### Data extraction

Two investigators (KPN and CYH) independently extracted the data from all eligible publications. Any differences among evaluators were resolved through discussion with a third investigator (ST). A standardized pre-piloted data extraction sheet was used. The extracted information included: (1) Baseline features: First author's name, year of publication, country, recruitment period, sample size, patient's age, gender (male), hydronephrosis status, follow-up time, and details regarding intervention and control; (2) Pathologic outcomes: tumor grade, tumor stage; (3) Survival outcomes: OS, CSS, RFS, IVRFS, MFS, and hazard ratios (HRs) with 95% confidence intervals (CIs) of OS and CSS in univariate or multivariate Cox analyses for comparing surgical techniques: KSS vs. RNU; (4) Functional outcomes: preoperative eGFR, postoperative eGFR, and changes in eGFR.

### Study quality assessment

The quality of included studies was independently evaluated by two authors (LQZ and SS) using the Newcastle-Ottawa Scale (NOS) (4), which includes three domains: selection of the study population, comparability of the groups, and ascertainment of the outcome. High-quality articles were identified as those with NOS scores ranging from 6 to 9, whereas scores ranging from 0 to 5 were considered to indicate poor quality.

### Statistical analysis

Oncological outcomes were primarily assessed using available HRs from univariable or multivariate analyses and their corresponding 95% CIs and 5-year survival endpoints. When 5-year survival endpoints were not available, we applied the formula described by Parmar et al. (5). We used the software Engauge Digitizer version 4.1 to calculate values derived from published Kaplan-Meier curves.

Mean changes in eGFR were used to assess the surgery-related alterations in renal function. Mean preoperative and postoperative eGFR values were recorded, along with their standard deviations (SDs), to evaluate the weighted mean difference.

Continuous and dichotomous variables were considered: inverse variance weighted mean difference (WMD) was used to summarize continuous variables, while the Mantel-Haenszel test was used to calculate odds ratios (ORs) with 95% CIs for binary values. Meta-analyses of pooled data were conducted using a fixed-effects or random-effects model. Statistical heterogeneity was assessed using the Chi-square test and I^2^ test. The I^2^ values ranges from 0% to 100%, with higher values indicating a greater degree of heterogeneity. If I^2^ > 50%, significant between-study heterogeneity was present, and the random-effect model was used. Two-tailed p < 0.05 was considered statistically significant. The data were analyzed with Review Manager (RevMan) 5.3, which was developed by the Cochrane Collaboration and Stata 17 software (Stat Corp, College Station, TX, USA).

## Results

### Baseline characteristics of population study

**Figure 1:**
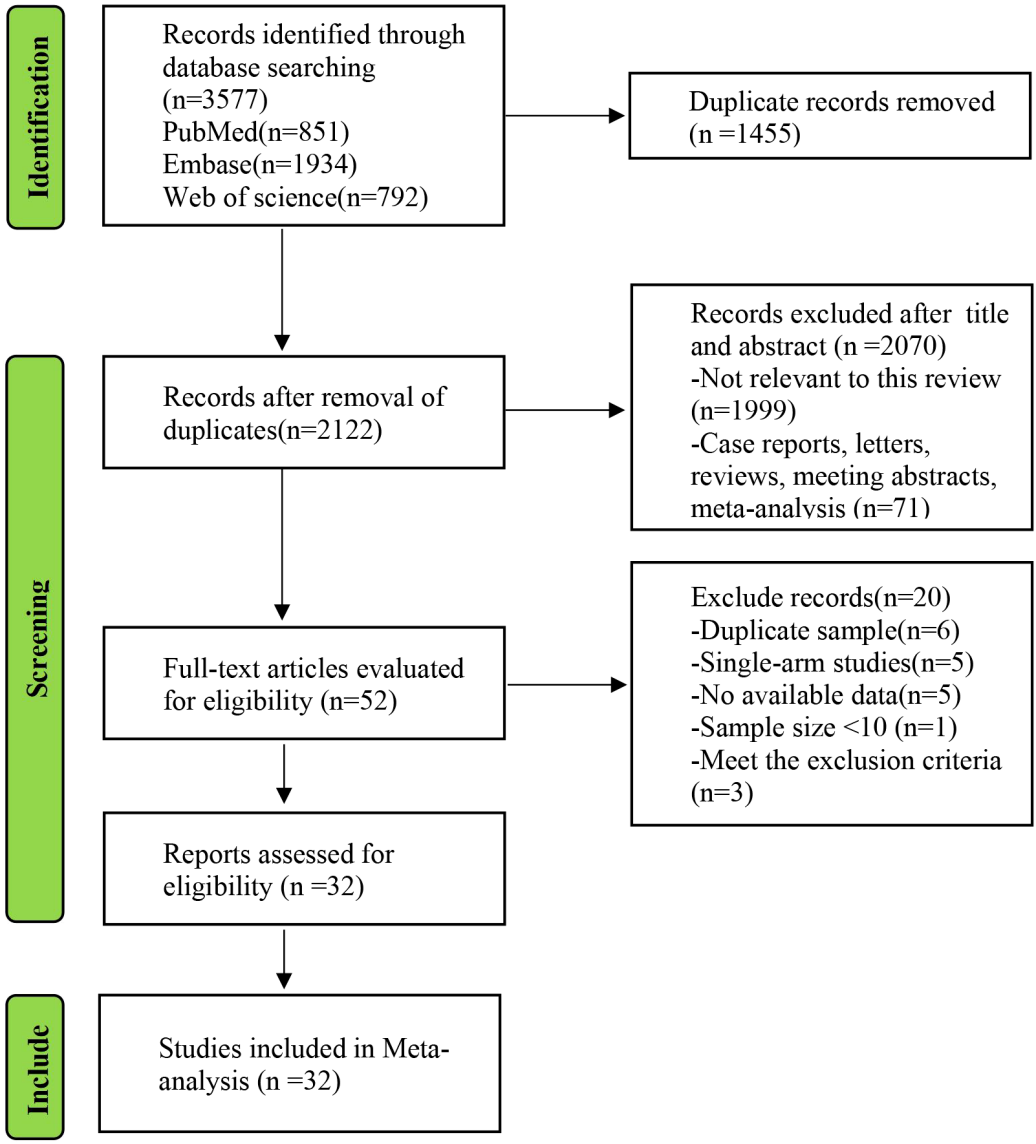
PRISMA flow diagram of the study process. PRISMA, Preferred Reporting Items for Systematic review and Meta-analysis.

The full process of the systematic literature review is shown in **Figure 1**. According to the PRISMA search strategy, no randomized controlled trials were available. Thirty-one retrospective studies and one prospective study, comprising a total of 21615 patients (7048 in the KSS group and 14567 in the RNU group), were ultimately included in the meta-analysis (**Table 1**) (6–37). Among these 32 articles, 10 focused on endoscopic management (EM), which included ureteroscopy surgery and percutaneous nephroscopy surgery, compared to RNU (6, 7, 10, 13, 14, 16, 28, 29, 34, 35), 19 studies compared SU to RNU (9, 11, 12, 15, 17, 18, 20–27, 30, 32, 33, 36, 37), and 3 involved comparisons among EM, SU, and RNU (8, 19, 31). The oncological characteristics and eGFR changes associated with surgery of the included studies are shown in **Supplementary Table 1**. The assessments of the NOS are presented in **Supplementary Table 2**, and the results showed that all the studies were of high quality with scores ranging from 6 to 8.

**Table 1:** Overall characteristics of the included studies.

Author/ Years	Country	Procedures	Study Period	Study Design	Centers	Patients (n)		Mean Age(yr)		Mean/Median follow-up (mo)		SQ
						KSS	RNU	KSS	RNU	KSS	RNU	
Rouprêt (6)	France	EM	1990-2004	Ret	Multiple	43	54	/	/	50.9 ± 22.9	61.0 ± 19	6
Gadzinski (7)	USA	EM	1996-2004	Ret	Single	34	62	/	/	/	/	7
Bin (8)	USA	EM,SU	2000-2010	Ret	Single	27	33	/	74.5	Median 29.0	Median 29.0	7
Colin (9)	France	SU	1995-2009	Ret	Multiple	52	416	70.3	69.1	Median 26.0	Median 26.0	7
Grasso (10)	USA	EM	1996-2011	Pro	Single	80	80	72.1	71.9	/	30.4(1-185.3)	8
Silberstein (11)	USA	SU	1994-2009	Ret	Single	33	87	68.6	70.9	/	/	7
Bagrodia (12)	USA	SU	/	Ret	Multiple	81	754	/	/	Median 34.0	Median 34.0	6
Cutress (13)	UK	EM	1991-2011	Ret	Single	59	70	/	/	56.0 ± 4.7	59.6 ± 5.0	7
Fajkovic (14)	Austria	EM	1996-2012	Ret	Multiple	20	178	71.9	68.9	Mean 20.4	Mean 20.4	6
Fukushima (15)	Japan	SU	/	Ret	Multiple	43	86	/	/	/	/	7
Hoffman (16)	Israel	EM	2000-2010	Ret	Single	25	22	63.9	74.2	26.0 (12–126)	57.0 (12–149)	6
Hung (17)	Taiwan	SU	2004-2010	Ret	Single	35	77	69.3	66.7	48.3 ± 26.97	43.8 ± 20.64	7
Pedrosa (18)	USA	SU	1999-2012	Ret	Single	35	96	67.8	70.8	/	/	6
Seisen (19)	France	EM,SU	2004-2013	Ret	/	176	128	69.5	67.3	37.2 ± 37.2	35.1 ± 28.5	7
Fang (20)	China	SU	2003-2016	Ret	Single	53	78	68.5	64.7	50.9 ± 20.28	55.3 ± 26.6	7
Kato (21)	Japan	SU	2004-2016	Ret	Single	12	14	72.5	73.7	48.5 (7–148)	46.9 (6–122)	7
Zhang (22)	China	SU	2005-2016	Ret	Single	47	109	/	/	34.5 (5–135)	59.0 (1–135)	6
Huang (23)	China	SU	2011-2016	Ret	Single	24	39	71.9	64.9	22.4 ± 17.57	26.1 ± 21.77	6
Jia (24)	China	SU	2000-2014	Ret	Single	40	179	69.6	70.1	63.7 ± 3.4	58.1 ± 8.1	7
Li (25)	China	SU	2007-2014	Ret	Single	73	182	67.6	66.7	/	/	7
Abrate (26)	Italy	SU	2003-2013	Ret	Multiple	26	67	71.7	72.7	25.8(13.2-35.8)	27.4(17.4-37.6)	7
Kim (27)	Korea	SU	2008-2016	Ret	Single	40	40	68.1	68.7	13.4 ± 14.1	29.7 ± 11.5	7
Shen (28)	Taiwan	EM	2004-2018	Ret	Single	23	42	67.4	67.3	/	/	6
Shenhar (29)	Israel	EM	2000-2018	Ret	Single	24	37	70.7	71.5	60.2 ± 47.0	57.0 ± 37.44	7
Chen (30)	Taiwan	SU	2005-2021	Ret	Single	30	39	68.3	67.5	Mean 33.0	Mean 68.0	6
Kim (31)	Korea	EM,SU	2011-2019	Ret	Single	62	646	67.7	68.3	65.8 ± 37.2	68.2 ± 36.7	8
Paciotti (32)	USA	SU	2004-2015	Ret	NCDB	4045	9016	71.9	71.8	/	/	8
Qiu (33)	China	SU	2004-2015	Ret	SEER	647	647	/	/	/	/	7
Tsujino (34)	Japan	EM	1990-2022	Ret	Multiple	35	108	72.8	70.4	Median 17.0	Median 39.0	6
Ye (35)	China	EM	2004-2020	Ret	SEER	397	397	/	/	/	/	8
Lee (36)	Korea	SU	2011-2020	Ret	Single	46	127	64.8	66.8	39.8 ± 21.4	53.3 ± 34.8	7
Ślusarczyk (37)	Poland	SU	2004-2018	Ret	SEER	694	694	/	/	/	/	7

EM, Endoscopic management; SU, Segmental Ureterectomy; KSS, Kidney-sparing surgery; RNU, Radical nephroureterectomy; Ret, Retrospective; Pro: Prospective; SQ, Study quality according to Newcastle-Ottawa Scale; SEER, Surveillance, Epidemiology, and End Results; NCDB, The National Cancer Database.

The survival outcomes in the included studies are described in **Table 2**. No statistically significant differences were found in terms of age or gender (**Supplementary Figure 1A, B**). However, some significant differences were observed between the KSS and RNU groups. Patients treated with KSS were less likely to have hydronephrosis (OR,0.45; 95%CI, 0.27-0.76; P=0.003) (**Supplementary Figure 1C**) and were more likely to have low-grade tumors (OR,1.80; 95%CI,1.39-2.33; P<0.0001) compared with those treated with RNU (**Supplementary Figure 1D**). Additionally, patients in the KSS group also presented with a lower tumor stage of ≤pT1 (OR, 1.69; 95% CI, 1.33-2.16; P < 0.0001) (**Supplementary Figure 1E**).

**Table 2:** Survival outcomes of the included studies.

Author/Years	OS (%)		OS: HR[95% CI] (KSS vs RNU)	CSS (%)		CSS: HR[95% CI] (KSS vs RNU)	year RFS (%)		year IVRFS (%)		year MFS (%)	
	KSS	RNU		KSS	RNU		KSS	RNU	KSS	RNU	KSS	RNU
Rouprêt (6)*	/	/	/	5-y:80.4	5-y:84.0	/	5-y:71.7	5-y:75.3	/	/	/	/
Gadzinski (7)	5-y:62.5	5-y:58.7	/	5-y:96.4	5-y:79.3	/	/	/	/	/	5-y:94.2	5-y:73.8
Bin (8)	/	/	/	/	/	/	/	/	/	/	/	/
Colin (9)	/	/	/	5-y:81.0	5-y:78.3	Un:0.99 [0.47-2.08] Mu:1.26[0.58-2.72]	5-y:28.1	5-y:46.8	/	/	5-y:76.3	5-y:73.9
Grasso (10)	5-y:60.1	5-y:58.0	/	5-y:71.5	5-y:64.0	/	/	/	/	/	5-y:69.3	5-y:60.0
Silberstein (11)	3-y:80.0	3-y:83.1	/	3-y:89.0	3-y:87.0	/	/	/	/	/	/	/
Bagrodia (12)			/	5-y:67.5	5-y:72.1	Un:1.06[0.65-1.73]	5-y:69.4	5-y:75.9	/	/	/	/
Cutress (13)	5-y:64.1	5-y:74.8	Mu:1.82[0.77-4.30]	5-y:85.6	5-y:92.1	Mu:3.26[0.83-12.8]	/	/	5-y:66.8	5-y:44.3	/	/
Fajkovic (14)	5-y:45.0	5-y:76.0	/	5-y:67.0	5-y:91.0	/	/	/	/	/	/	/
Fukushima (15)	/	/	/	5-y:86.0	5-y:76.0	Un:1.17 [0.53-2.60] Mu:1.61[0.66-3.07]	5-y:84.0	5-y:69.0	/	/	/	/
Hoffman (16)	3-y:68.3	3-y:95.2	/	/	/	/	/	/	/	/	/	/
Hung (17)	/	/	/	5-y:89.5	5-y:82.5	/	/	/	5-y:61.0	5-y:56.9	5-y:89.6	5-y:75.7
Pedrosa (18)	3-y:57.5	3-y:55.3	Un:0.93[0.56-1.56]	3-y:67.6	3-y:69.2	Un:1.10[0.57-2.10]	3-y:35.0	3-y:48.6	/	/	/	/
Seisen (19)	5-y:79.3	5-y:73.5	/	5-y:86.7	5-y:87.4	/	/	/	5-y:54.8	5-y:46.7	/	/
Fang (20)	5-y:55.4	5-y:72.1	/	5-y:81.9	5-y:82.8		/	/	5-y:58.8	5-y:55.0	5-y:79.9	5-y:76.2
Kato (21)	5-y:77.8	5-y:60.1	Un:0.63[0.15-2.65]	5-y:87.5	5-y:71.9	Un:0.70[0.11-4.34]	5-y:34.4	5-y:50.0	/	/	5-y:80.8	5-y:73.5
Zhang (22)	5-y:80.3	5-y:76.4	/	/	/	Mu:0.53[0.25-1.16]	/	/	/	/	/	/
Huang (23)	3-y:72.1	3-y:83.8	/	3-y:79.0	3-y:87.6	/	3-y:50.3	3-y:68.0	3-y:68.3	3-y:86.3	3-y:100	3-y:92.4
Jia (24)	5-y:61.7	5-y:61.0	Un:1.55 [0.87-2.79] Mu:1.31[0.72-2.38]	5-y:66.8	5-y:64.1	Un:1.39 [0.77-2.50] Mu:1.61[0.63-2.13]	/	/	5-y:46.4	5-y:45.4	5-y:90.3	5-y:96.0
Li (25)	/	/	/	3-y:74.0	3-y:72.6	Un:1.01 [0.65-1.57] Mu:1.71[0.61-4.78]	/	/	/	/	/	/
Abrate (26)	5-y:46.8	5-y:52.0	/	/	/	/	/	/	/	/	/	/
Kim (27)	3-y:71.5	3-y:87.5	/	3-y:82.6	3-y:93.0	/	3-y:73.2	3-y:68.2	3-y:36.9	3-y:42.3	/	/
Shen (28)	5-y:94.5	5-y:94.6	Un:1.79[0.35-9.13]	/	/	/	/	/	5-y:75.2	5-y:55.8	/	/
Shenhar (29)	5-y:85.0	5-y:84.0	/	5-y:89.0	5-y:92.0	/	/	/	/	/	5-y:81.0	5-y:84.0
Chen (30)	5-y:39.3	5-y:64.5	/	5-y:60.1	5-y:78.3	/	5-y:44.0	5-y:67.2	5-y:67.2	5-y:77.1	/	/
Kim (31)	5-y:68.0	5-y:60.7	/	5-y:74.0	5-y:71.7	Un:0.97[0.53-1.76]	5-y:40.5	5-y:37.6	/	/	5-y:67.0	5-y:64.1
Paciotti (32)	5-y:53.1	5-y:52.6	Un:0.98[0.93-1.04]	/	/	/	/	/	/	/	/	/
Qiu (33)	5-y:51.0	5-y:52.5	Un:0.99[0.88-1.11]	5-y:57.3	5-y:57.6	Un:1.05[0.90-1.20]	/	/	/	/	/	/
Tsujino (34)	3-y:80.4	3-y:90.7	Un:2.42[0.63-9.28]	/	/		/	/	/	/	/	/
Ye (35)	5-y:65.3	5-y:80.3	Mu:1.63[1.37-1.94]	5-y:83.2	5-y:94.0	Mu:2.23[1.67-2.97]	/	/	/	/	/	/
Lee (36)	3-y:88.8	3-y:83.1	/	3-y:91.2	3-y:93.6	/	3-y:79.8	3-y:73.5	3-y:50.4	3-y:54.5	/	/
Ślusarczyk (37)	5-y:48.9	5-y:48.2	Mu:1.03[0.90-1.18]	5-y:63.9	5-y:62.3	Mu:1.03[0.87-1.23]	/	/	/	/	/	/

*Oncological outcomes of the article “Rouprêt 2006” was low-grade group; OS, Overall survival; CSS, Cancer specific survival; RFS, Recurrence free survival; IVRFS, Intravesical recurrence free survival; MFS, Metastasis-free survival; HR, Hazard ratio; CI, Confidence interval; Un, Univariate Cox regression analysis; Mu, Multivariable Cox regression analysis.

### Oncologic outcomes

**Figure 2:**
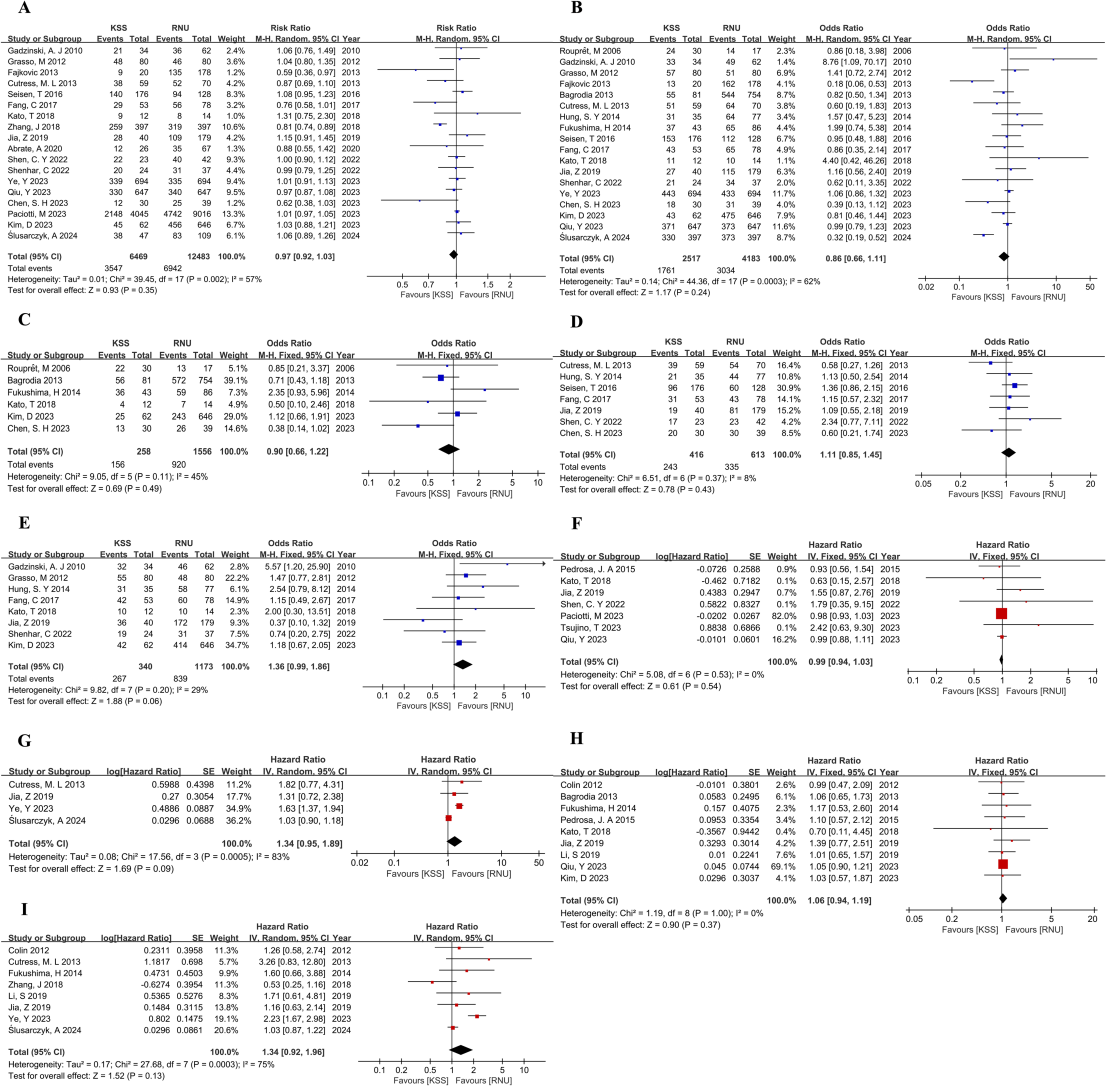
Forest plot of meta-analyses comparing oncologic outcomes between the KSS and RNU groups: **(A)** 5-year OS; **(B)** 5-year CSS; **(C)** 5-year RFS; **(D)** 5-year IVRFS; **(E)** 5-year MFS; **(F)** HR for OS from univariate analyses; **(G)** HR for OS from multivariate analyses; **(H)** HR for CSS from univariate analyses; **(I)** HR for CSS from multivariate analyses.

Our investigation revealed no significant differences in terms of 5-year OS, 5-year CSS, 5-year RFS, 5-year IVRFS, and 5-year MFS between the KSS and RNU groups, as shown in **Figures 2A–E**. This finding was confirmed by both univariate and multivariate Cox regression analysis. The surgical treatment-related hazard ratios (HRs) for KSS versus RNU from univariate analyses for OS were HR, 0.99; 95% CI, 0.94-1.03; P=0.54 (**Figure 2F**). From multivariate analyses for OS, the HR was 1.34; 95% CI, 0.95-1.89; P=0.09 (**Figure 2G**). Univariate analyses for CSS showed an HR of 1.06 (95% CI, 0.94-1.19; P=0.37) (**Figure 2H**), while multivariate analyses for CSS showed an HR of 1.34 (95% CI, 0.92-1.96; P=0.13) (**Figure 2I**). Similarly, the observed differences were not statistically significant.

### Subgroup analysis

**Figure 3:**
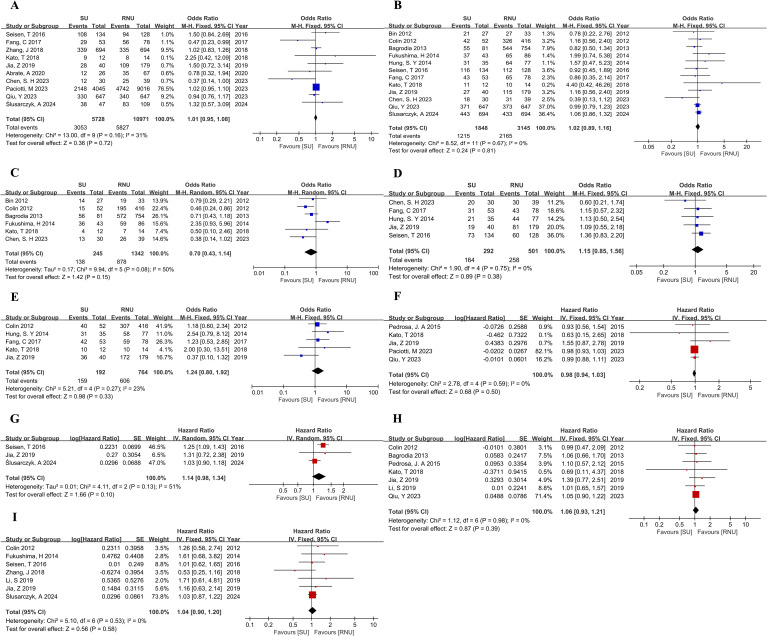
Forest plot of meta-analyses comparing oncologic outcomes between the SU and RNU groups: **(A)** 5-year OS; **(B)** 5-year CSS; **(C)** 5-year RFS; **(D)** 5-year IVRFS; **(E)** 5-year MFS; **(F)** HR for OS from univariate analyses; **(G)** HR for OS from multivariate analyses; **(H)** HR for CSS from univariate analyses; **(I)** HR for CSS from multivariate analyses.

To reduce heterogeneity, we conducted a comprehensive comparison of survival outcomes between different treatment groups. Subgroup analysis was performed on SU versus RNU and EM versus RNU. In terms of pathologic outcomes between the SU and RNU groups, no statistically significant differences were found in tumor grade (OR,1.24; 95%CI, 0.99-1.55; P=0.06)(**Supplementary Figure 2A**). However, the SU group had a lower tumor stage of ≤pT1 (OR, 1.47; 95% CI, 1.19-1.93; P =0.0004)(**Supplementary Figure 2B**), which was statistically significant. The 5-year OS, 5-year CSS, 5-year RFS,5-year IVRFS, and 5-year MFS showed no significant differences between SU and RNU groups. The same results for OS and CSS were observed in both univariate and multivariate Cox regression analyses between SU and RNU groups (**Figure 3**).

**Figure 4:**
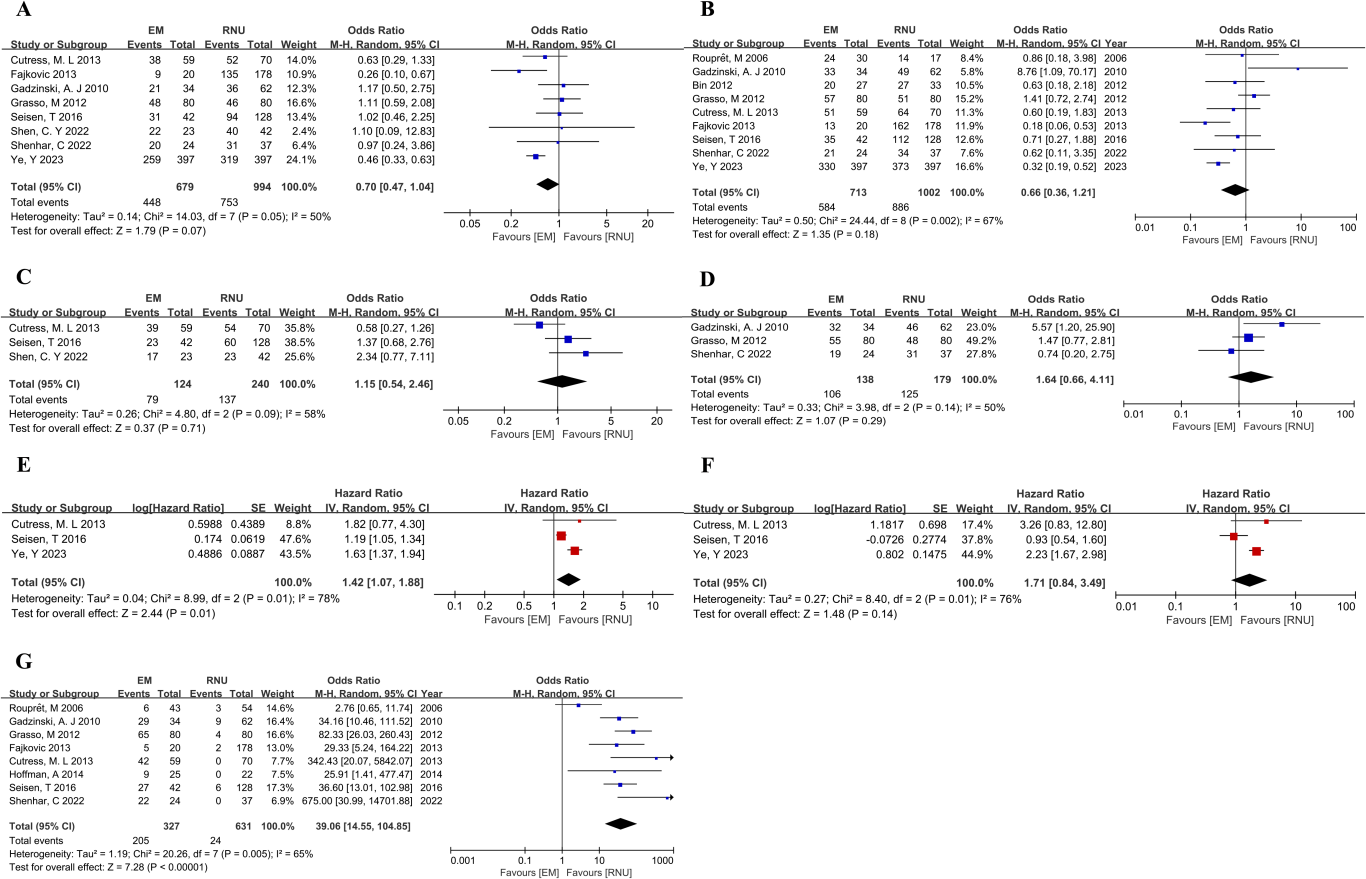
Forest plot of meta-analyses comparing oncologic outcomes between the EM and RNU groups: **(A)** 5-year OS; **(B)** 5-year CSS; **(C)** 5-year IVRFS; **(D)** 5-year MFS; **(E)** HR for OS from multivariate analyses; **(F)** HR for CSS from multivariate analyses; **(G)** Upper tract recurrence rate.

In the comparison between the EM and RNU groups, significant differences were also observed in pathologic outcomes. The EM group had more low-grade tumors (OR,4.67; 95%CI,1.77-12.32; P=0.002) and low-stage tumors (≤pT1) (OR, 2.65; 95% CI, 1.31-5.37; P=0.007) (**Supplementary Figure 3**). There were no significant differences in 5-year OS, 5-year CSS, 5-year IVRFS, and 5-year MFS between the EM and RNU groups, as depicted in **Figure 4A–D**. However, in multivariate Cox regression analysis, the HR for OS was 1.42 (95%CI,1.07-1.88; P=0.01) between the EM and RNU groups, indicating a statistically significant difference and suggesting had worse overall survival outcomes for endoscopic excision compared to RNU (**Figure 4E**). There was no statistically significant difference in CSS (HR,1.71; 95%CI,0.84-3.48; P=0.14) in multivariate analysis (**Figure 4F**). Moreover, the upper tract recurrence rate was calculated and found to be significantly higher in the EM group compared to the RNU group (OR,39.06; 95%CI,14.55-104.85; P<0.00001) (**Figure 4G**).

### Renal function outcomes

**Figure 5:**
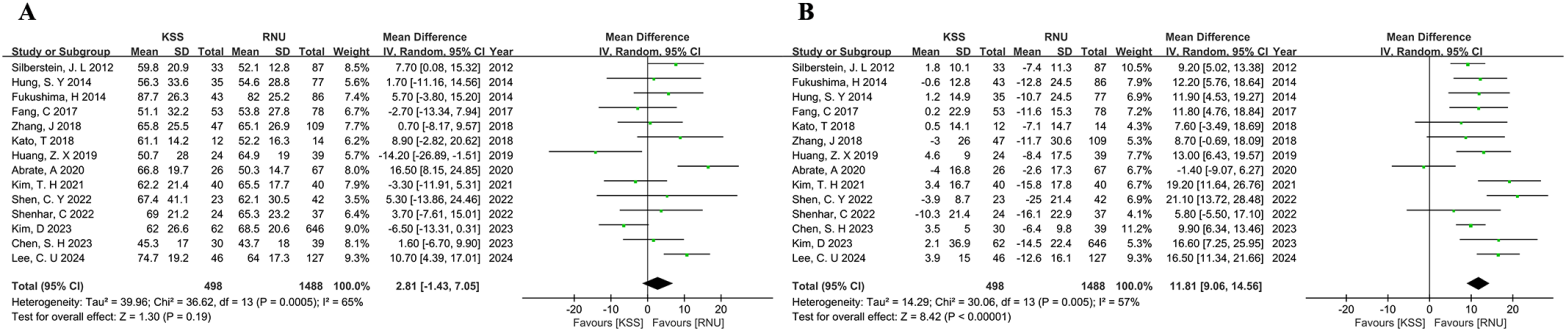
Forest plot of meta-analyses comparing renal function between the KSS and RNU groups: **(A)** Preoperative eGFR; **(B)** Weighted mean changes in eGFR.

No statistically significant difference was found in preoperative eGFR between the KSS and RNU groups (WMD, 2.81 ml/min/1.73 m^2^; 95%CI,-1.43-7.05;P=0.19) (**Figure 5A**). Howere, significant differences were observed in the weighted mean changes in eGFR between the two groups. In patients treated with KSS, postoperative eGFR increased by 0.4 ml/min/1.73m^2^, whereas it decreased by 11.4ml/min/1.73 m^2^ in the RNU group (WMD, 11.81 ml/min/1.73m^2^; 95%CI, 9.06-14.56; P<0.0001) (**Figure 5B**). That indicates that patients treated with KSS experienced significantly better preservation of renal function compared to those treated with RNU.

### The sensitivity analysis and publication bias

**Figure 6:**
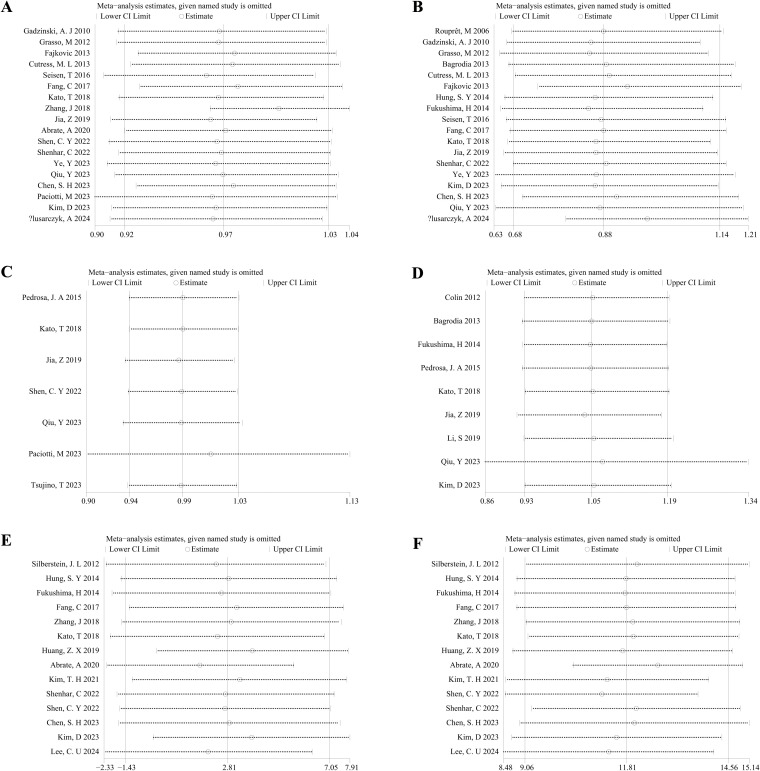
Sensitivity analysis of the association between the KSS and RNU groups: **(A)** 5-year OS; **(B)** 5-year CSS; **(C)** HR for OS from univariate analyses; **(D)** HR for CSS from univariate analyses; **(E)** Preoperative eGFR; **(F)** Weighted mean changes in eGFR.

A sensitivity analysis was performed on the 5-year OS, 5-year CSS, HR for OS from univariate analyses, HR for CSS from univariate analyses, preoperative eGFR, and weighted mean changes in eGFR (as depicted in **Figure 6**) between the KSS and RNU groups. The significance of the pooled comparison between the two groups was not influenced by the removal of any single study, indicating that the results of our meta-analysis were stable.

**Figure 7:**
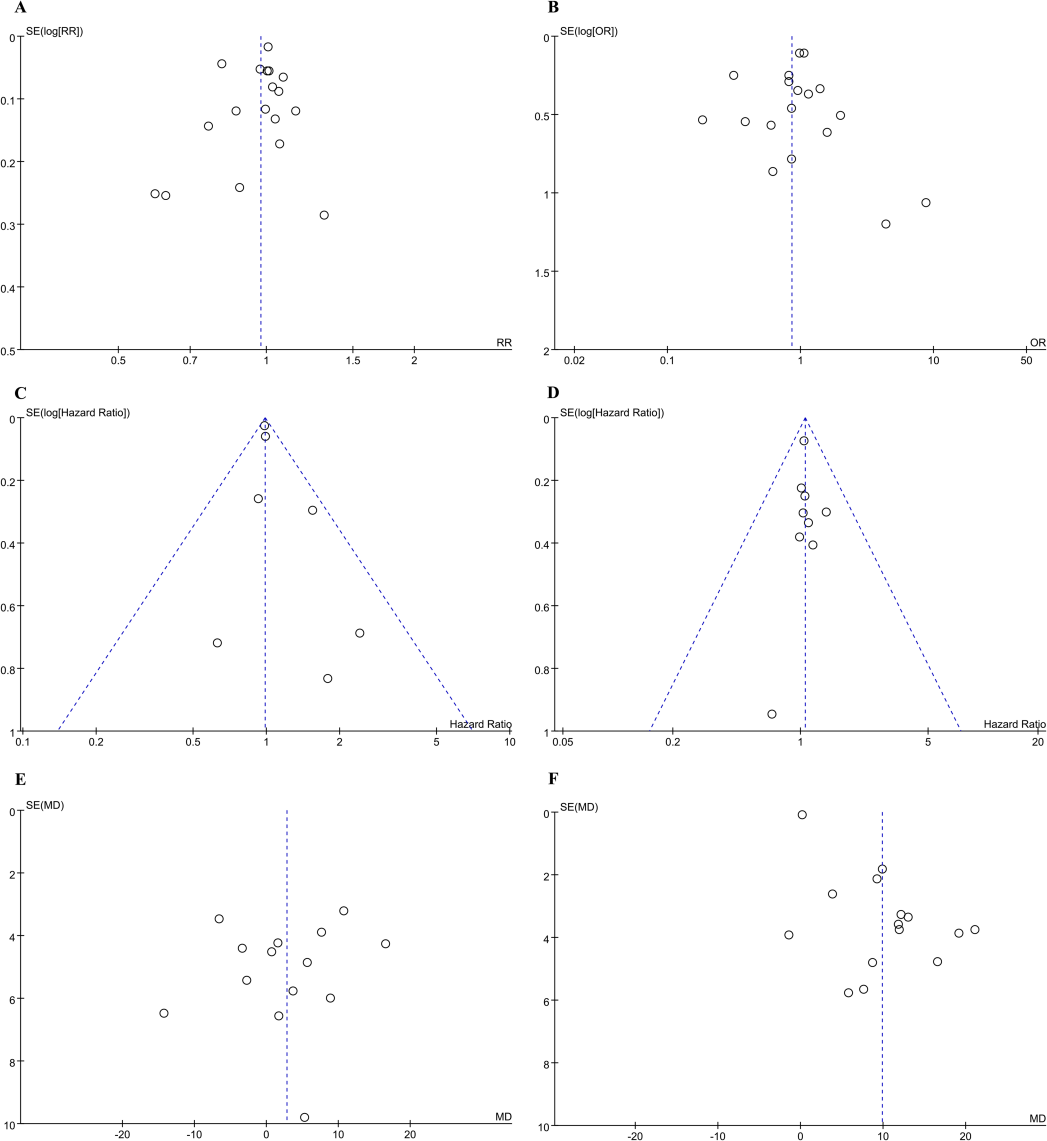
Funnel plot for the evaluation of potential publication bias between the KSS and RNU groups: **(A)** 5-year OS; **(B)** 5-year CSS; **(C)** HR for OS from univariate analyses; **(D)** HR for CSS from univariate analyses; **(E)** Preoperative eGFR; **(F)** Weighted mean changes in eGFR.

The funnel plots were used to assess the publication bias of the included studies, and no significant publication bias was detected between KSS and RNU groups regarding 5-year OS, 5-year CSS, HR for OS from univariate analyses, HR for CSS from univariate analyses, preoperative eGFR, weighted mean changes in eGFR (**Figure 7**).

## Discussion

In recent years, KSS has been increasingly performed to avoid complications associated with RNU and to theoretically preserve postoperative renal function. However, the assumptions regarding the comparative outcomes between KSS and RNU are predominantly based on heterogeneous data derived from small retrospective cohort studies, which are considered to provide a low level of evidence in the current literature (38, 39). To address this gap, we conducted a systematic review and meta-analysis to compare oncological and renal function outcomes between KSS and RNU. Our analysis was based on data obtained from all available comparative studies, with additional subgroup analyses conducted to examine differences between SU and RNU, as well as between EM and RNU.

### Oncologic outcomes (KSS VS. RNU)

In our meta-analysis, KSS demonstrated feasible efficacy regarding oncological outcomes. Specifically, there were no significant differences in the 5-year OS, 5-year CSS, 5-year RFS, 5-year IVRFS, and 5-year MFS between the KSS and RNU groups. This finding was consistent across both univariate and multivariate Cox regression analyses for OS and CSS. In the survival outcomes mentioned above, it is generally believed that the IVRFS of the KSS group is often inferior to that of the RNU group due to the complete bladder cuff excision performed in the RNU group. However, our study found no significant difference in 5-year IVRFS between the two groups, which may be attributed to the lower grade (18, 19, 31, 32, 34) or lower stage (11, 18, 19, 26, 31, 34, 40) of disease in the KSS group. Additionally, patients undergoing RNU were more likely to present with preoperative hydronephrosis (11, 41) and more advanced disease at the outset, which may have contributed to a reduced survival rate. This patient selection bias may have potentially skewed the results. Despite these considerations, based on the outcomes of our study, KSS can be a viable treatment option for patients with lower-grade and lower-stage UTUC.

### Subgroup analysis (SU VS. RNU)

Since KSS encompasses both the EM and SU approaches, we conducted a subgroup analysis between SU and RNU to reduce selection bias. Patients treated with ureteroureterostomy or ureteral reimplantation for ureteral tumors are collectively referred to as undergoing SU. Our study found no significant differences in the 5-year OS, 5-year CSS, 5-year RFS,5-year IVRFS, and 5-year MFS between the SU and RNU groups. This was further supported by the results of univariate and multivariate Cox regression analyses for OS and CSS, which showed no significant difference between the two groups. According to the EAU guidelines, SU is recommended only for imperative cases or highly selected patients with low-risk UTUC, due to the potential increased risk of ipsilateral upper urinary tract recurrence and CSS compared to radical extirpation surgery (3). However, our study demonstrated no statistically significant difference in RFS, IVRFS, and CSS between the SU and RNU groups. Even though all included trials in our meta-analysis involved high-grade tumors, the survival outcomes showed no significant differences between SU and RNU. This suggests that SU can be a valid option for patients with high-grade ureteric urothelial cancer, particularly for those with distally located tumors in the ureter. SU could provide complete removal of tumors, lymph nodes, and possibly invaded tissues. Based on our findings, SU can be safely performed regardless of tumor grade for patients with distal ureteral tumors, without increasing patient mortality.

### Subgroup analysis (EM VS. RNU)

Over the past decade, advancements in material and laser technology have led to an increased adoption of endoscopic management for UTUC by many surgical teams (42). EM, which includes percutaneous and ureteroscopic resection, fulguration, or ablation, has been widely applied (19). Numerous case-control studies have suggested that endoscopic management can be recommended as an alternative to nephroureterectomy for low-risk or superficial UTUC, with no observed impact on survival (16, 28, 29, 34). However, our meta-analysis revealed that the local recurrence rate (OR, 39.06) was substantially higher in the EM group compared to the RNU group. Upper tract recurrence is an inevitable consequence of endoscopic treatment, regardless of tumor grade. The high local recurrence rate necessitates repeated endoscopic treatments or delayed nephrectomy, imposing an increased mental, physical, and financial burden on patients (35). Furthermore, in multivariate Cox regression analysis, the HR for OS was found to be higher in the EM group, indicating a shorter OS for this group and multiple studies have reached the same conclusion (19, 35). Another limitation of the ureteroscopic treatment for UTUC includes the potential for under grading on initial biopsy and the inability to fully assess the extent of UTUC (35). Despite this, no significant differences were observed in the 5-year OS, 5-year CSS, 5-year IVRFS, 5-year MFS, and CSS in multivariate analysis.

### Renal function outcomes

The primary advantage of KSS is the preservation of kidney function, which can lead to better outcomes in renal function and increased eligibility for adjuvant chemotherapy (27). In our meta-analysis, we observed that postoperative eGFR increased by 0.4ml/min/1.73m^2^ in patients treated with KSS, while it decreased by 10.4ml/min/1.73 m^2^ in the RNU group (WMD,10.78ml/min/1.73m^2^). This indicates that preserving the ipsilateral renal unit results in significantly less reduction in renal function compared to RNU. The improved renal function following KSS is likely due to the resection of a partially or completely obstructing ureteral tumor, thereby allowing for enhanced ipsilateral renal function (36). Another study found that the greatest difference in renal function was observed during follow-up at 3 months after intervention, but no significant difference was seen at 2and 5 years after intervention. This also indicates that, at least within the first 2 years, the renal function in the KSS group is superior to that of the RNU group (43). Moreover, patients who underwent KSS were more likely to be candidates for adjuvant chemotherapy because of the preservation of eGFR (44). More importantly, the preservation of the renal unit is crucial for enhancing life expectancy (29). Thus, our results suggest that patients may experience better postoperative renal function after KSS compared to RNU, thereby avoiding the unnecessary risk of postoperative dialysis, which can significantly impact the quality of life.

To our knowledge, this is the only meta-analysis comparing KSS with RNU, and it is also the most up-to-date systematic review and meta-analysis comparing SU with RNU, as well as EM with RNU. However, our study is limited by its retrospective design and the heterogeneity in definitions, inclusion criteria, therapies, follow-up periods, reporting methods, and surgeon expertise. These factors make the study susceptible to selection and reporting biases. Nevertheless, this limitation is inherent to the field of UTUC. In the future, there is a need for more multicenter, randomized controlled trials with large sample sizes and high quality. Ideally, these studies would provide more accurate data, including detailed information on tumor size, location, stage, and grade.

## Conclusions

Our systematic review and meta-analysis support the notion that KSS yields similar oncological outcomes compared to RNU, albeit with the caveat that tumor characteristics were not equally balanced between the two groups. KSS can be considered a viable treatment option for patients with low-grade and low-stage UTUC. Furthermore, we observed that renal function preservation is significantly better after KSS compared to RNU. Although EM is part of KSS and is associated with a higher local recurrence rate and a shorter OS in multivariate analysis, it may necessitate repeated surgical interventions or eventual acceptance of delayed RNU. On the other hand, the distinct benefits of SU include the en-bloc resection of the ureteral tumor with surrounding soft tissue and lymph node dissection. Thus, we believe that SU could be a better alternative for the treatment of ureteral tumors compared with ureteroscopic approaches. Additionally, renal function preservation is significantly better after SU when compared to RNU. These findings suggest that SU should be considered a first-line treatment for low-grade UTUC of the ureter and may also be appropriate for selected cases of high-grade UTUC.

## Data Availability

The datasets presented in this study can be found in online repositories. The names of the repository/repositories and accession number(s) can be found in the article/**Supplementary Material**.

## Supplementary material

Supplementary Figure 1 Forest plot of meta-analyses comparing baseline characteristics between the KSS and RNU groups: **(A)** Age; **(B)** Gender; **(C)** Hydronephrosis; **(D)** Low-grade tumor; **(E)** Tumor Stage≤pT1.

Supplementary Figure 2 Forest plot of meta-analyses comparing pathologic outcomes between the SU and RNU groups: **(A)** Low-grade tumor; **(B)** Tumor Stage≤pT1.

Supplementary Figure 3 Forest plot of meta-analyses comparing pathologic outcomes between the EM and RNU groups: **(A)** Low-grade tumor; **(B)** Tumor Stage≤pT1.
