# Supplementary material for: Kidney sparing surgery versus radical nephroureterectomy in upper tract urothelial carcinoma: a meta-analysis and systematic review
Source: Front Oncol. 2025 Apr 2;15:1448079. doi: 10.3389/fonc.2025.1448079 (PMC11999840; doi:10.3389/fonc.2025.1448079)
Supplement: Supplementary file 1 [file Table1.docx]

Supplementary Table 1: Oncological characteristics and eGFR changes of the included studies

| Author / Years | Hydronephrosis, n (%) | | KSS | | | RNU | | | KSS | | | RNU | | | Changes in eGFR | |
| --- | --- | --- | --- | --- | --- | --- | --- | --- | --- | --- | --- | --- | --- | --- | --- | --- |
|  | KSS | RNU | G1/LG | G2 | G3/HG | G1/  LG | G2 | G3/HG | ≤T1 | T2 | ≥T3 | ≤T1 | T2 | ≥T3 | KSS(M ±SD) | RNU(M ±SD) |
| Rouprêt (6) | / | / | 30 |  | 13 | 17 |  | 37 | 36 | 6 | 1 | 25 | 17 | 12 | / | / |
| Gadzinski (7) | / | / | / | / | / | / | / | / | / | / | / | / | / | / | / | / |
| Bin (8) | / | / | 12 |  | 15 | 3 |  | 30 | 17 | 7 | 3 | 18 | 3 | 12 | / | / |
| Colin (9) | / | / | 3 | 23 | 26 | 23 | 144 | 249 | 34 | 12 | 6 | 224 | 41 | 151 | / | / |
| Grasso (10) | / | / | 66 |  | 14 | 23 |  | 57 | / | / | / | / | / | / | / | / |
| Silberstein (11) | 13(39.4%) | 69(79.3%) | 8 |  | 25 | 18 |  | 69 | 25 | 5 | 3 | 39 | 25 | 23 | 1.8±10.1 | -7.4±11.3 |
| Bagrodia (12) | / | / | 36 |  | 45 | 171 |  | 583 | 56 | 9 | 16 | 348 | 136 | 270 | / | / |
| Cutress (13) | / | / | 34 | 19 | 6 | 8 | 46 | 16 | 59 | 0 | 0 | 70 | 0 | 0 | / | / |
| Fajkovic (15) | / | / | 14 |  | 3 | 134 |  | 44 | 20 | 0 | 0 | 178 | 0 | 0 | / | / |
| Fukushima (15) | 26(60.5%) | 64(74.4%) | 6 | 22 | 15 | 9 | 37 | 40 | 29 | 5 | 9 | 38 | 20 | 28 | -0.6±12.8 | -12.8±24.5 |
| Hoffman (16) | / | / | 25 |  | 0 | 22 |  | 0 | / | / | / | / | / | / | -4.0 | -10.0 |
| Hung (17) | / | / | 5 |  | 30 | 9 |  | 68 | 24 | | 11 | 62 | | 15 | 1.2 ±14.9 | -10.7±24.5 |
| Pedrosa (18) |  |  | 16 |  | 19 | 15 |  | 81 | 20 | 7 | 7 | 48 | 13 | 25 | / | / |
| Seisen (19) | 99(56.3%) | 72(56.3%) | 100 |  | 76 | 41 |  | 87 | 104 | 23 | 7 | 77 | 39 | 12 | / | / |
| Fang (20) | 29(54.7%) | 45(57.7%) | 32 |  | 21 | 48 |  | 30 | 25 | 28 | | 27 | 51 | | 0.2±22.9 | -11.6±15.3 |
| Kato (21) | / | / | 6 |  | 6 | 7 |  | 7 | 6 | 3 | 3 | 9 | 1 | 4 | 0.5±14.1 | -7.1±14.7 |
| Zhang (22) | / | / | 21 |  | 26 | 37 |  | 71 | 15 | 32 | | 28 | 80 | | -3.0±26.0 | -11.7±30.6 |
| Huang (23) | 19(79.2%) | 35(89.7%) | 6 |  | 18 | 8 |  | 31 | 9 | 9 | 6 | 11 | 13 | 15 | 4.6±9.0 | -8.4±17.5 |
| Jia (24) | / | / | 0 | 23 | 17 | 10 | 82 | 87 | 18 | 12 | 10 | 60 | 79 | 40 | / | / |
| Li (25) | 56(76.7%) | 157(86.3%) | / | / | / | / | / | / | 35 | 25 | 7 | 82 | 48 | 44 | / | / |
| Abrate (26) | 20(76.9%) | 60(89.6%) | 4 | 12 | 10 | 2 | 26 | 39 | 18 | 6 | 0 | 31 | 16 | 20 | -4.0±16.8 | -2.6±17.3 |
| Kim (27) | / | / | 2 | 15 | 23 | 3 | 21 | 16 | 16 | 13 | 11 | 21 | 11 | 8 | 3.4±16.7 | -15.8±17.8 |
| Shen (28) | 7(30.4%) | 33(78.6%) | 6 |  | 17 | 6 |  | 36 | 23 | 0 | 0 | 42 | 0 | 0 | -3.9±8.7 | -25.0±21.4 |
| Shenhar (29) | / | / | / | / | / | / | / | / | 23 | 0 | 0 | 36 | 0 | 0 | -10.3±21.4 | -16.1±22.9 |
| Chen (30) | 30(100%) | 39(100%) | 1 |  | 29 | 5 |  | 34 | 13 | 9 | 8 | 13 | 16 | 10 | 3.5±5.0 | -6.4±9.8 |
| Kim (31) | / | / | 17 |  | 38 | 105 |  | 526 | 41 | 9 | 12 | 328 | 77 | 241 | 2.1±36.9 | -14.5±22.4 |
| Paciotti (32) | / | / | 684 |  | 1254 | 1291 |  | 3138 | 1704 | 338 | 356 | 3454 | 741 | 714 | / | / |
| Qiu (33) | / | / | 115 |  | 532 | 123 |  | 524 | / | / | / | / | / | / | / | / |
| Tsujino (34) | / | / | 16 |  | 19 | 23 |  | 85 | 35 | 0 | 0 | 108 | 0 | 0 | 1.1±0.3 | 0.9±0.8 |
| Ye (35) | / | / | 186 |  | 73 | 188 |  | 92 | 397 | 0 | 0 | 397 | 0 | 0 | / | / |
| Lee (36) | / | / | 25 | | 18 | 56 | | 69 | 23 | 12 | 11 | 50 | 32 | 45 | 3.9±15.0 | −12.6±16.1 |
| Ślusarczyk (37) | / | / | 126 |  | 568 | 125 |  | 569 | 435 | | 259 | 435 | | 259 | / | / |

LG: Low-grade; HG: High-grade; eGFR: estimated glomerular filtration rate (ml/min/1.73 m^2^).
