# Supplementary material for: Kidney sparing surgery versus radical nephroureterectomy in upper tract urothelial carcinoma: a meta-analysis and systematic review
Source: Front Oncol. 2025 Apr 2;15:1448079. doi: 10.3389/fonc.2025.1448079 (PMC11999840; doi:10.3389/fonc.2025.1448079)
Supplement: Supplementary file 2 [file Table2.docx]

Supplementary Table 2: Results of quality assessment using the Newcastle-Ottawa Scale for case-control studies

| Study | Selection | | | | Comparability | Exposure | | | Scores |
| --- | --- | --- | --- | --- | --- | --- | --- | --- | --- |
|  | Is the Case Definition Adequate? | Representativeness of the Cases | Selection of Controls | Definition of Controls | Comparability of Cases and Controls on the Basis of the Design or Analysis | Ascertainment of Exposure | Same method of ascertainment for cases and controls | Non-Response rate |  |
| Rouprêt (6) | ☆ | ☆ | ☆ | ☆ |  | ☆ | ☆ |  | 6 |
| Gadzinski (7) | ☆ | ☆ | ☆ | ☆ | ☆ | ☆ | ☆ |  | 7 |
| Bin (8) | ☆ | ☆ | ☆ | ☆ | ☆ | ☆ | ☆ |  | 7 |
| Colin (9) | ☆ | ☆ | ☆ | ☆ | ☆ | ☆ | ☆ |  | 7 |
| Grasso (10) | ☆ | ☆ | ☆ | ☆ | ☆ | ☆ | ☆ | ☆ | 8 |
| Silberstein (11) | ☆ | ☆ | ☆ | ☆ |  | ☆ | ☆ | ☆ | 7 |
| Bagrodia (12) | ☆ | ☆ | ☆ | ☆ |  | ☆ | ☆ |  | 6 |
| Cutress (13) | ☆ | ☆ | ☆ | ☆ |  | ☆ | ☆ | ☆ | 7 |
| Fajkovic (14) | ☆ | ☆ | ☆ | ☆ |  | ☆ | ☆ |  | 6 |
| Fukushima (15) | ☆ | ☆ | ☆ | ☆ | ☆ | ☆ | ☆ |  | 7 |
| Hoffman (16) | ☆ | ☆ | ☆ | ☆ |  | ☆ | ☆ |  | 6 |
| Hung (17) | ☆ | ☆ | ☆ | ☆ | ☆ | ☆ | ☆ |  | 7 |
| Pedrosa (18) | ☆ | ☆ | ☆ | ☆ |  | ☆ | ☆ |  | 6 |
| Seisen (19) | ☆ | ☆ | ☆ | ☆ | ☆ | ☆ | ☆ |  | 7 |
| Fang (20) | ☆ | ☆ | ☆ | ☆ | ☆ | ☆ | ☆ |  | 7 |
| Kato (21) | ☆ | ☆ | ☆ | ☆ | ☆ | ☆ | ☆ |  | 7 |
| Zhang (22) | ☆ | ☆ | ☆ | ☆ |  | ☆ | ☆ |  | 6 |
| Huang (23) | ☆ | ☆ | ☆ | ☆ |  | ☆ | ☆ |  | 6 |
| Jia (24) | ☆ | ☆ | ☆ | ☆ | ☆ | ☆ | ☆ |  | 7 |
| Li (25) | ☆ | ☆ | ☆ | ☆ | ☆ | ☆ | ☆ |  | 7 |
| Abrate (26) | ☆ | ☆ | ☆ | ☆ | ☆ | ☆ | ☆ |  | 7 |
| Kim (27) | ☆ | ☆ | ☆ | ☆ | ☆ | ☆ | ☆ |  | 7 |
| Shen (28) | ☆ | ☆ | ☆ | ☆ |  | ☆ | ☆ |  | 6 |
| Shenhar (29) | ☆ | ☆ | ☆ | ☆ | ☆ | ☆ | ☆ |  | 7 |
| Chen (30) |  | ☆ | ☆ | ☆ | ☆ | ☆ | ☆ |  | 6 |
| Kim (31) | ☆ | ☆ | ☆ | ☆ | ☆ | ☆ | ☆ | ☆ | 8 |
| Paciotti (32) | ☆ | ☆ | ☆ | ☆ | ☆☆ | ☆ | ☆ |  | 8 |
| Qiu (33) | ☆ | ☆ | ☆ | ☆ | ☆ | ☆ | ☆ |  | 7 |
| Tsujino (34) | ☆ | ☆ | ☆ | ☆ |  | ☆ | ☆ |  | 6 |
| Ye (35) | ☆ | ☆ | ☆ | ☆ | ☆ | ☆ | ☆ | ☆ | 8 |
| Lee (36) | ☆ | ☆ | ☆ | ☆ | ☆ | ☆ | ☆ |  | 7 |
| Ślusarczyk (37) |  | ☆ | ☆ | ☆ | ☆☆ | ☆ | ☆ |  | 7 |
