# Supplementary figures and images for: Kidney sparing surgery versus radical nephroureterectomy in upper tract urothelial carcinoma: a meta-analysis and systematic review
Source: Front Oncol. 2025 Apr 2;15:1448079. doi: 10.3389/fonc.2025.1448079 (PMC11999840; doi:10.3389/fonc.2025.1448079)

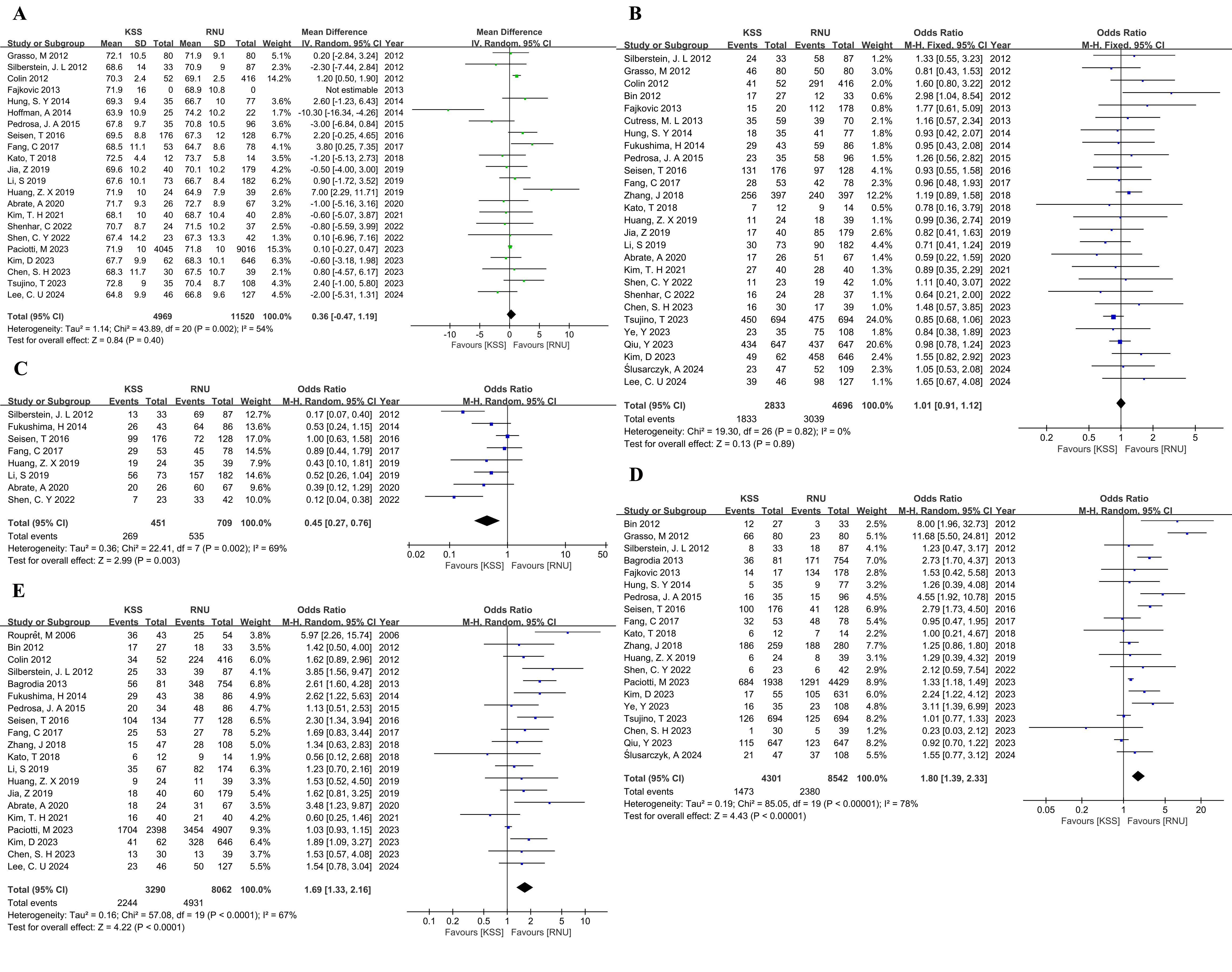

Supplement: Supplementary Figure 1 — Forest plot of meta-analyses comparing baseline characteristics between the KSS and RNU groups: (A) Age; (B) Gender; (C) Hydronephrosis; (D) Low-grade tumor; (E) Tumor Stage≤pT1. [file Image1.tif]

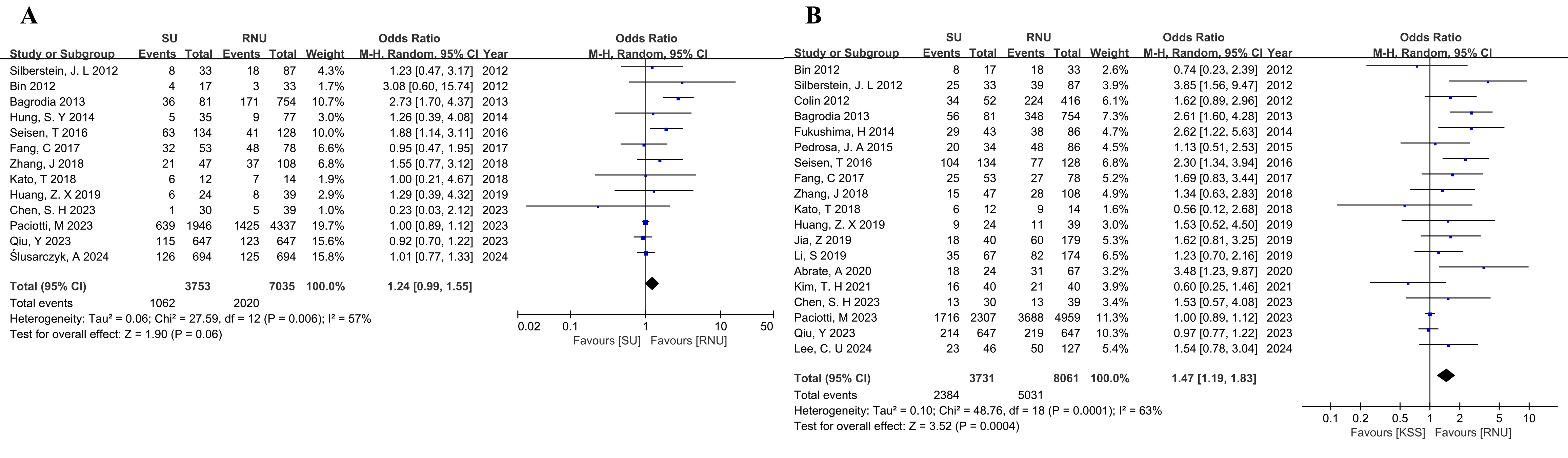

Supplement: Supplementary Figure 2 — Forest plot of meta-analyses comparing pathologic outcomes between the SU and RNU groups: (A) Low-grade tumor; (B) Tumor Stage≤pT1. [file Image2.tif]

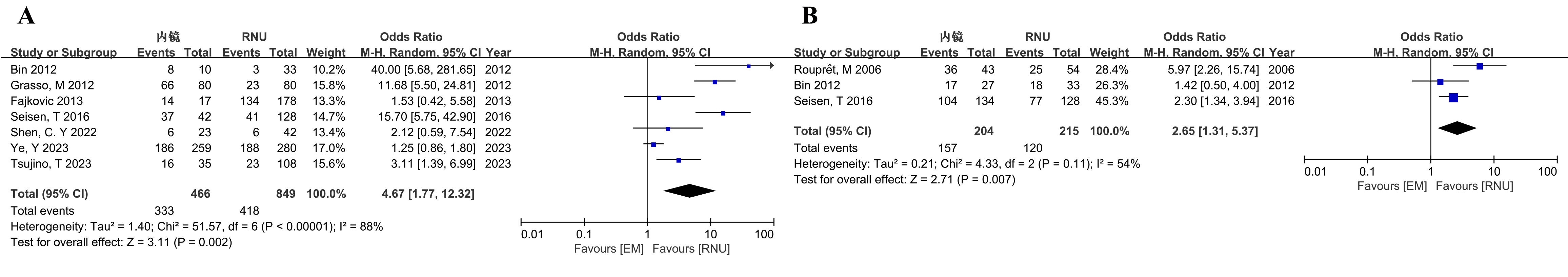

Supplement: Supplementary Figure 3 — Forest plot of meta-analyses comparing pathologic outcomes between the EM and RNU groups: (A) Low-grade tumor; (B) Tumor Stage≤pT1. [file Image3.tif]
